# Variety in Food

**Published:** 1891-06

**Authors:** 


### VARIETY IN FOOD.

Many people fall into the mistake of supposing that, because they take a certain number of meals daily, and keep their stomachs in constant employment, they have satisfied the needs of the body. A doctor was recently called in to attend upon a seamstress who was suffering from weakness and general depression. "What kind of food have you been living upon?" asked he of his patient. "Oh! my food has been all right," she replied. "I have lived mostly on bread and butter for a long time, and I am sure there is no harm in that." "My dear woman," exclaimed the doctor, "you have explained the secret of your illness. Your body has been starving on that diet, and I am surprised you are no worse." The functions which the body has to perform are many and various, and the supply of food must be complex to meet its requirements. The internal organs are constantly at work, and therefore wasting; the body must be kept warm to a large extent by food.

Then there is the wear and tear of the system which an ordinary day, work involves. Whether one's life be one of comparative ease, or filled with arduous toil, this variety of diet must be maintained, and flesh forming, heat giving, and starchy matter must be taken in proper proportions. With this fact before us, it is a duty incumbent upon all to understand the nature and composition of food, and its fitness for the nourishment of the human frame. Under natural conditions, the appetite is a fair guide in the selection of materials, but our methods of cooking and seasoning are so contrary to reason that its power in that direction is not of much use. Food should also be regulated in quantity by the amount of physical labor to be performed. An artisan or farm laborer, who spends Saturday afternoon and Sunday in resting after a week of exacting toil, does not require quite so much food during his leisure. Yet, in the majority of instances, he eats more, and, as a consequence, is in worse trim on returning to his work than he was when he left it. When the food supply is kept up, and mechanical labor diminished, the system is burdened with a surplus of matter which the tissues cannot dispose of. The strain then falls to the internal organs, and, when this happens, indigestion, headache, and several other disorders make themselves known. Over-feeding and disregard for the true requirements of the human frame in its diverse experiences have been the cause of much physical discomfort. We have on several occasions given in tabulated form, the nutritive value of the various dishes in common use, with their chemical properties and adaptation to the various elements which the system requires for the preservation of vigorous health. We cannot reasonably hope to be healthy ourselves, or that our children will develop and become strong, unless our food is adapted to our physical needs.

